# Comparative Genomic Analysis of Capsule-Producing Black Yeasts *Exophiala dermatitidis* and *Exophiala spinifera*, Potential Agents of Disseminated Mycoses

**DOI:** 10.3389/fmicb.2020.00586

**Published:** 2020-04-08

**Authors:** Yinggai Song, Nickolas Menezes da Silva, Vinicius Almir Weiss, Duong Vu, Leandro F. Moreno, Vania Aparecida Vicente, Ruoyu Li, G. Sybren de Hoog

**Affiliations:** ^1^Department of Dermatology and Venerology, Peking University First Hospital, Beijing, China; ^2^Research Center for Medical Mycology, Peking University, Beijing, China; ^3^National Clinical Research Center for Skin and Immune Diseases, Beijing, China; ^4^Microbiology, Parasitology and Pathology Post-Graduation Program, Department of Pathology, Federal University of Paraná, Curitiba, Brazil; ^5^Center of Expertise in Mycology of Radboud University Medical Center/Canisius Wilhelmina Hospital, Nijmegen, Netherlands; ^6^Graduate Program in Bioprocess Engineering and Biotechnology, Federal University of Paraná, Curitiba, Brazil; ^7^Westerdijk Fungal Biodiversity Institute, Utrecht, Netherlands; ^8^Amsterdam Medical Center, Amsterdam, Netherlands

**Keywords:** black yeasts, comparative genomics, intraspecific variability, interspecific differences, virulence profiles, opportunists

## Abstract

The two black yeasts *Exophiala dermatitidis* and *Exophiala spinifera* that are clinically considered as the most virulent species potentially causing disseminated infections are both producing extracellular capsule-like material, are compared. In this study, 10 genomes of *E. spinifera* and *E. dermatitidis* strains, including both clinical and environmental isolates, were selected based on phylogenetic analysis, physiology tests and virulence tests, sequenced on the Illumina MiSeq sequencer and annotated. Comparison of genome data were performed between intraspecific and interspecific strains. We found capsule-associated genes were however not consistently present in both species by the comparative genomics. The prevalent clinical species, *E. dermatitidis*, has small genomes containing significantly less virulence-associated genes than *E. spinifera*, and also than saprobic relatives. Gene OG0012246 and Myb-like DNA-binding domain and SANT/Myb domain, restricted to two strains from human brain, was shared with the neurotropic species *Rhinocladiella mackenziei*. This study indicated that different virulence profiles existed in the two capsule-producing black yeasts, and the absence of consistent virulence-associated profiles supports the hypothesis that black yeasts are opportunists rather than primary pathogens. The results also provide the key virulence genes and drive the continuing research forward pathogen–host interactions to explore the pathogenesis.

## Introduction

Black yeasts in the ascomycete order Chaetothyriales are relatively frequent opportunistic agents of human disease. Species of Chaetothyriales producing budding cells in any stage of their life cycle are classified currently in the genus *Exophiala*. The Atlas of Clinical Fungi (de Hoog et al., 2019) lists 19 species that were proven to have been involved in infections of humans or cold-blooded vertebrates. Of these, *Exophiala dermatitidis* and *Exophiala spinifera* are the most common species in clinical settings, and are the only recurrent agents of severe, deep and disseminated infections in humans, then often with fatal outcome. In the past, patients were reportedly without significant underlying disease, but recent research has shown that such infections are mostly associated with an inherited defect in the dectin signaling pathway due to mutations in the *CARD9* gene (Lanternier et al., 2015).

Virulence of black yeasts has been attributed to the presence of melanin in cell walls (Taborda et al., 2008), but since all members of the order Chaetothyriales are consistently melanized, this does not explain the difference in infective ability between species. Thermotolerance, for which marked differences are noted between species, is another important factor: 11 out of 19 opportunistic *Exophiala* species are at least weakly tolerant of 37°C (de Hoog et al., 2019), with *E. dermatitidis* and *E. spinifera* having the most pronounced thermotolerance. However, de Hoog et al. (2011) described a ‘waterborne clade’ of mesophilic species in the genus, comprising species commonly infecting fish, frogs, toads or crabs, often at epidemic proportions (Vicente et al., 2012; Pearson et al., 2019; Saraiva et al., 2019). Consequently, black yeasts lacking thermotolerance are also able to cause vertebrate infection. Gostincar et al. (2018) suggested polyextremotolerance as a prerequisite for opportunism, highlighting the interplay of several, independent factors enabling growth under non-optimal conditions of animal tissue. The black yeasts and relatives mitigate external stress of, e.g., dryness and irradiation by melanin, and detrimental effects of toxin can be compensated by pathways of the cytochrome P450 (Moreno et al., 2018) aiding degradation and co-assimilation of monoaromatic hydrocarbons. This combination of vitality factors provides a wide array of survival strategies, and it may be hypothesized that species with the most pronounced development of such pathways may have a higher ability of tissue invasion. In addition, Yurlova and de Hoog (2002) noted that *E. dermatitidis* and *E. spinifera* are unique in producing extracellular capsule-like structures during early exponential growth of budding cells. This may further enhance opportunism. Both species are able to disseminate in susceptible patients with formation of cutaneous acanthosis, but a difference in clinical predilection has been noted: *E. dermatitidis* has a tendency of neurotropism, whereas *E. spinifera* seems to be somewhat osteotropic (Song et al., 2017). This suggests that opportunism in these fungi might be fine-tuned.

The aim of the present paper is to compare the genomes of the above opportunists in *Exophiala* with each other and with species with other types or with no opportunistic potentials, in search of genes that might play a role in the above described differences. Several genomes were sequenced of each species, enabling to compare the intra-species variability in the genome in general and in potential virulence genes.

## Materials and Methods

### Strains, DNA Extraction, and Genome Sequencing

To extract genomic DNA, fungal mycelia of *E. dermatitidis* strains CBS 109144, CBS 115663, CBS 120473, CBS 132758, CBS 132754, and CBS 578.76, and *E. spinifera* strains CBS 101539, CBS 116557, CBS 123469, CBS 126013, and CBS 131564 were harvested from fresh cultures on Sabouraud’s Glucose Agar (GSA), washed using sterile Tris-EDTA buffer (TE), pH 8.0 in 2 mL vol screw-capped tubes, and then resuspended in 500 ml TE buffer. Fungal cell walls were disrupted using 0.5 mm glass beads in a BioSpec Mini-Beadbeater-16 (BioSpec) for 5 min and cooled on ice for an additional 5 min. DNA solutions were separated using two phenol/chloroform (1:24, pH 8.0) extractions. DNA was then precipitated by isopropanol, washed with 70% ethanol, dried at room temperature, and resuspended in 35 mL TE buffer, pH 8.0. DNA quantity and quality were determined using Qubit (Invitrogen, Applied BioSystems), and an Agilent Bio Analyzer 2100 using a 1000 DNA Chip (Agilent). Cardinal growth temperatures were determined on 2% malt extract agar (MEA; Difco). Plates were incubated at 37, 40, 42, 45°C in the dark for 2 weeks; plates contained double quantities of medium and were sealed to prevent drying out. Colony diameters were measured for studied strains.

### Genome Sequencing

The extracted genome DNA of *E. spinifera* and *E. dermatitidis* strains were sequenced on the MiSeq (IlluminaTM, San Diego, CA, United States) sequencer using paired-end and mate-paired libraries. The library construction was done with Ion Plus Fragment Library Kit (Thermo Fisher ScientificTM) and Nextera XT (IlluminaTM) following the manufacturer instructions.

### Genome Assembly

Quality control of the reads was performed using FastQC v0.11.8^1^, and low-quality sequences were removed by BBMap^2^. High-quality reads were assembled by SPAdes v3.10.0 (Bankevich et al., 2012) using the respective reference genomes, *E. dermatitidis* CBS 525.76 = NIH/UT8656 and *E. spinifera* CBS 899.68, except to the *Exophiala phaeomuriformis*, that was made a *de novo* assembly. To finish the assemblies, the gaps were closed using FGAP (Piro et al., 2014). To identify *de novo* repeats across the genome assembly, the library produced by RepeatModeler was used as input for RepeatMasker v4.0.7. Genes were predicted by producing a training set using Genemark-ES v4.30 (Lomsadze et al., 2005). The predicted functional annotations and proteins it was performed by InterProScan v5.27-66.0 (Quevillon et al., 2005).

### Read Mapping and SNP Calling

For SNP calling, high-quality sequencing reads of each of the genomes were mapped against the reference genome of the reference strains *E. dermatitidis* CBS 525.76 = NIH/UT8656 and *E. spinifera* CBS 899.68 deposited at GenBank, respectively, by using Burrows Wheeler Aligner (BWA) v0.7.17-r1188 mem (Li and Durbin, 2009) and sorted to the bam format using SAMtools v1.7. They were then marked duplicated using Picard v1.8^3^ and indexed using SAMtools. Variants were identified using GATK HaplotypeCaller v3.4.9.0. SNP annotation was performed by VCFannotator^4^ to assess whether the SNP was found within an untranslated region, intron, or coding exon, and mutations were classified into synonymous (SYN), non-synonymous (NSY), read-through (RTH), and nonsense (STP).

### Ortholog Detection

The protein sequences of the 10 strains of the two species *E. dermatitidis* and *E. spinifera* were clustered using OrthoFinder v2.1.2 (Emms and Kelly, 2015) to determine which proteins were shared or specific by the two species. In addition, the protein sequences of the five *E. dermatitidis* strains (CBS 109144, CBS 115663, CBS 120473, CBS 132758, and CBS 578.76) were also clustered to see which proteins were shared between isolates from brain (CBS 578.76, CBS 120473) and environmental isolates (CBS 109144, CBS 115663, CBS 132758).

Possible relationships of analyzed gene families with invasion of cerebral versus bone tissue which are critical virulence genes for the understanding of black yeast virulence, were searched in the literature, with functional confirmation in the online InterPro database^5^. Relevant genes and domains were blasted against the *E. spinifera* and *E. dermatitidis* genomes.

## Results

### Data Quality and Identification

**FIGURE 1:**
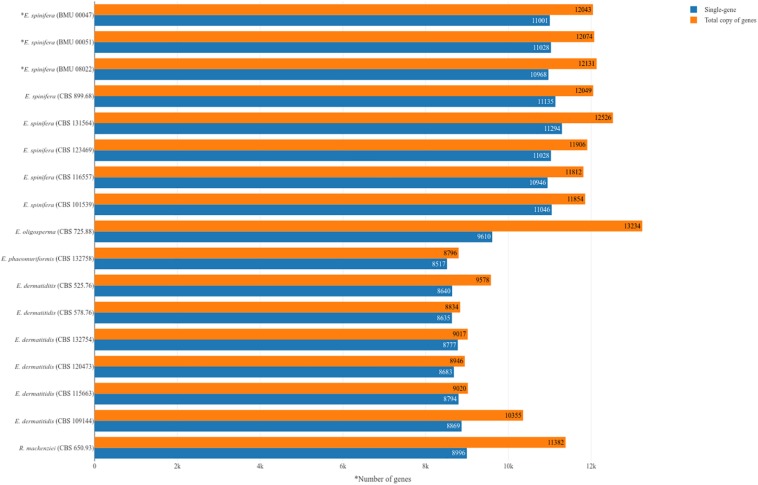
Numbers of genes in analyzed strains of *Exophiala spinifera* and *E. dermatitidis*, compared with reference genomes. ^∗^Strains analyzed with combined Illumina and PacBio approach; remaining strains analyzed with Illumina.

**FIGURE 2:**
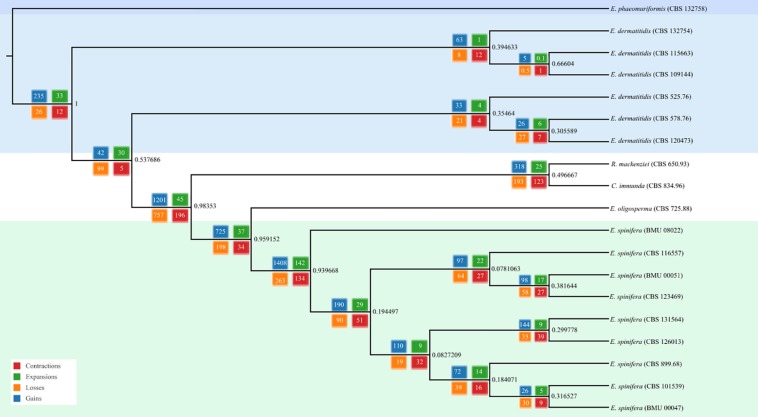
Phylogenomic tree based on whole genomic analysis using OrthoFinder; 5,374 single-copy orthogroups were detected, representing 41.86% of orthologous groups.

Genomes of five strains of each species were sequenced (Table 1). Different techniques were applied, i.e., Pacbio and Illumina. Comparison of genome data of the same strain of *E. spinifera*, BMU00047 (JAAABH000000000) with the reference genome (CBS 899.68, JYBY00000000.1) provided perfect match with Illumina, but some translocations were noted compared to PacBio data (Figure 1). The genomes assembled with PacBio had less gaps and had more copies of the same gene. However, when single-genes were counted without duplication, the results of genome assembly are very similar to those assembled with Illumina reads (Figure 1). The reads of the newly sequenced strains of the two black yeasts *E. dermatitidis* and *E. spinifera* were quality-controlled. Low-quality reads were removed. The size of the forward/reverse sequencing reads of these strains ranged from 400 to 700M. The number of scaffolds obtained for each genome is presented in Table 2. The high-quality forward/reverse sequencing reads were assembled using *de novo* assembly. The genomes had sizes ranging between 25.88 and 34.3M. The genomes of *E. dermatitidis* on average were smaller than those of *E. spinifera*. Intraspecific variability was evaluated using Orthofinder, for strains of *E. dermatitidis* and of *E. spinifera* separately. In total, 55,750 clustered genes were found. Percentages of genomic variation ranged from 0.2 to 8.6% (1,626 genes) in *E. dermatitidis* genomes, while for *E. spinifera*, with a total of 108,201 genes, variation ranged from 0.5 to 3.2% (2,052 genes). In the genomic dendrogram (Figure 2), *E. spinifera* strains demonstrated a higher degree of branching, which is explained by a lower percentage (71.6%) of single-copy orthogroups (*n* = 8,979), while for *E. dermatitidis* this was 85.7% (*n* = 7,640). Estimation of gene gains and losses shows that *E. spinifera* has higher divergence than *E. dermatitidis*, with averages of 268.13 gains and 74.75 losses, versus 21 gains and 31 losses per speciation event, respectively. Compared to sibling species *E. phaeomuriformis* of *E. dermatitidis* and *E. oligosperma* of *E. spinifera*, considerable gene gains and expansions were noted (Figure 2), which deviated significantly (3109.8 genes on average) from intra-specific changes. Gene dynamics also differed between the species of study, i.e., on average 2111 genes (σ 314.3 in shared genes and 608.1 in specific genes) in *E. dermatitidis*, and on average 4107.8 (σ 121.18 in shared genes and 55.9 in specific genes) in *E. spinifera*.

**TABLE 1:** Metadata of strains sequenced in this study, and reference isolates.

Strain ID	Species	Geography	Source	GenBank accession
CBS 101539	*E. spinifera*	Colombia	Soil	JAAAJH000000000
CBS 116557	*E. spinifera*	Thailand	Pine apple	JAAAJG000000000
CBS 126013	*E. spinifera*	Brazil	Shell of babasu coconut	JAAAJF000000000
CBS 131564	*E. spinifera*	Thailand	Human toenail	JAAAJE000000000
CBS 123469	*E. spinifera*	China	Human skin	JAAAJD000000000
BMU 08022R	*E. spinifera*	China	Human skin (CARD9 deficient)	JAAABF000000000
BMU 00051R	*E. spinifera*	China	Bark	JAAABG000000000
BMU 00047R	*E. spinifera*	Colombia	Soil	JAAABH000000000
CBS 899.68TR	*E. spinifera*	United States	Human nasal granuloma	JYBY00000000
CBS 725.88TR	*E. oligosperma*	Germany	Human sphenoid abscess	JYCA01000000
CBS 132758R	*E. phaeomuriformis*	Turkey	Dishwasher	JAAAJI000000000
CBS 109144	*E. dermatitidis*	Netherlands	Turkish bath	WXYG00000000
CBS 132754	*E. dermatitidis*	Turkey	Bathtub	JAAAJK000000000
CBS 578.76	*E. dermatitidis*	Japan	Human brain	JAAAJJ000000000
CBS 115663	*E. dermatitidis*	Qatar	Endotracheal aspirate	JAAAJM000000000
CBS 120473	*E. dermatitidis*	United States	Human brain	JAAAJL000000000
CBS 525.76TR	*E. dermatitidis*	Japan	Human sputum	AFPA00000000.1
CBS 650.93R	*Rhinocladiella mackenziei*	Saudi Arabia	Human brain	JYBU00000000.1

*T = ex-type strain; R = published genomes included for comparison.*

**TABLE 2:** Genomes information of *E. dermatitidis, E. spinifera and E. phaeomuriformis*.

	Genome size	Number of scaffolds	Number of reads	GC content (%)	Number of genes	Assembly completeness (BUSCO)	Repeated sequences (simple repeats)	Repeated sequences (low complexity)
***E. dermatitidis***								
CBS 109144	29.6M	2094	21,487,952	51.18%	10,355	99.0%	0.92%	0.07%
CBS 115663	26.8M	68	20,912,714	51.40%	9,02	99.3%	0.89%	0.07%
CBS 120473	26.4M	245	19,003,776	51.61%	8,946	97.7%	0.88%	0.07%
CBS 132754	26.8M	326	11,165,600	51.41%	9,017	99.3%	0.89	0.07%
CBS 578.76	26.2M	219	20,397,964	51.60%	8,834	99.00%	0.90	0.07%
***E. spinifera***								
CBS 101539	32.9M	133	19,532,882	51.56%	11,854	97.7%	0.83%	0.08%
CBS 116557	32.5M	109	22,950,634	51.81%	11,812	97.3%	0.82%	0.08%
CBS 123469	32.4M	243	14,594,162	51.85%	11,906	98.0%	0.81%	0.08%
CBS 126013	32.6M	117	12,808,484	51.81%	11,806	98.0%	0.81%	0.08%
CBS 131564	34.3M	906	17,069,282	51.60%	12,526	98.3%	0.90%	0.07%
***E. phaeomuriformis***								
CBS 132758	25.86	97	85,740,696	51.60	8796	99.3%	0.88%	0.07%

**FIGURE 3:**
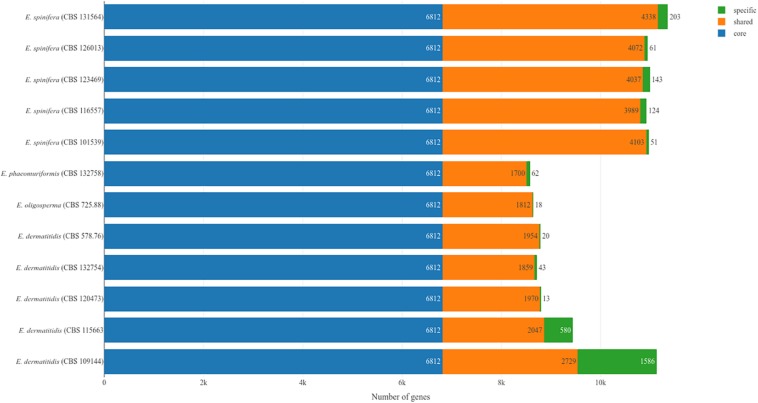
Core and accessory genomes of *E. spinifera* and *E. dermatitidis*, showing the number of unique genes in each strain.

The core genomes of 6,812 genes were determined by comparing the shared genes in all *Exophiala* species sequenced to date (Figure 3). The accessory genomes of *E. spinifera* were considerably larger than those of *E. dermatitidis*, but CBS 115663 and particularly CBS 109144 in the latter species deviated considerably from remaining strains by having 580 and 1,586 unique genes, respectively. The use of long reads provides better resolution, which is particularly significant for repetitive regions of the genome. Whole-genome comparison of all genomes of the two species with reference genomes of *E. phaeomuriformis* (nearest neighbor of *E. dermatitidis*), *E. oligosperma* (nearest neighbor of *E. spinifera*), *Cladophialophora immunda* (saprobic hydrocarbon-assimilating species) and *Rhinocladiella mackenziei* (opportunistic neurotropic species) was based on detection of 5,374 single-copy orthogroups representing 41.86% of orthologous groups. The genomic dendrogram (Figure 2) showed correct clustering of all genomes, but with significant intra-specific variability, which in *E. spinifera* was twice that of *E. dermatitidis*.

### Protein Prediction and SNP Calling

Protein sequences of the new sequenced strains were predicted using the homology-based predictor InterProScan v5.27-66.0 (Quevillon et al., 2005). The numbers of the obtained genes for each of the strain are given in Table 2. Protein sequences of the 10 genomes of the two species were clustered to detect orthologs using OrthoFinder v2.1.2 (Emms and Kelly, 2015). A total of 12,690 clusters was detected. Among these, 7,291 clusters had protein sequences shared by *E. dermatitidis* and *E. spinifera*; 1,456 clusters were represented in the *E. dermatitidis* proteome only, and 3,253 clusters were exclusively present in *E. spinifera*.

In order to detect genomic variants, the high-quality sequencing reads of all genomes of the two species were aligned with the respective reference genomes, *E. dermatitidis* CBS 525.76 = NIH/UT8656 and *E. spinifera* CBS 899.68. The total numbers of genomic variants and non-synonymous SNPs of the *E. dermatitidis* and *E. spinifera* genomes are given in Table 3. The numbers of non-synonymous SNPs in *E. dermatitidis* varied from 8,887 to 30,126, and in *E. spinifera* from 17,978 to 43,282. The two brain-associated isolates of *E. dermatitidis*, CBS 528.76 and CBS 120473, shared two clusters containing genes with unique SNPs. The first cluster contained the genes HMPREF1120_03262 of *E. dermatitidis* and Z518_05210 of *R. mackenziei* that was linked to benzaldenhyde dehydrogenase. The second cluster contained genes HMPREF1120_08762 of *E. dermatitidis* and Z518_06196 of *R. mackenziei*.

**TABLE 3:** Synonymous and non-synonymous SNP numbers of *E. dermatitidis* (CBS 525.76) and *E. spinifera* (CBS 899.68), compared with reference genomes.

*E. dermatitidis*	CBS 109144	CBS 115663	CBS 120473	CBS 132754	CBS 578.76
Number of SNPs	163,572	160,112	94,043	151,141	47,898
Number of non-syn SNPs	30,126	29,542	17,643	11,602	8,887
Number of unique non-syn SNPs	599	807	8618	19	3435
Number of transversions	64,693	65,859	35,208	49,645	18,794
Number of transitions	124,089	126,220	68,215	96,706	36,049

***E. spinifera***	**CBS 101539**	**CBS 116557**	**CBS 123469**	**CBS 126013**	**CBS 131564**

Number of SNPs	94,675	212,255	242,380	196,728	174,304
Number of non-syn SNPs	17,978	37,075	43,282	35,904	31,316
Number of unique non-syn SNPs	6863	15009	3487	1323	8
Number of transversions	32,874	73,413	83,343	67,764	58,902
Number of transitions	60,654	132,290	150,710	123,030	108,083

### Metabolism of Aromatic Compounds

**FIGURE 4:**
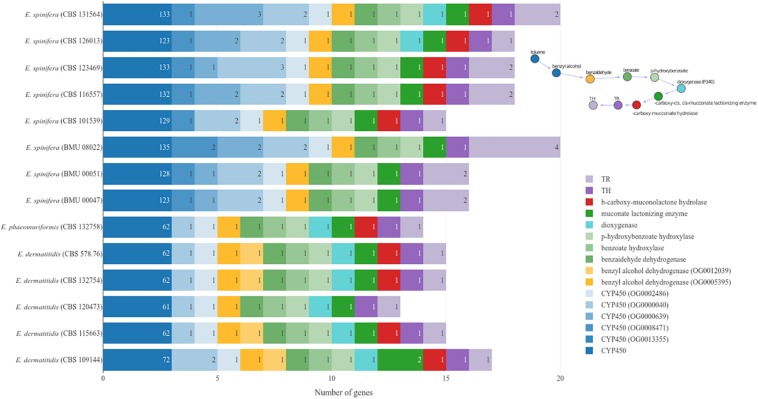
Genes involved in pathways for the metabolism of aromatic compounds.

To identify the presence of genes involved in pathways for the degradation of monoaromatic hydrocarbons (e.g., benzene, toluene, ethylbenzene, xylene, styrene, and other volatile pollutants), the genomes of reference strains of *E. dermatitidis* (CBS 525.76 = NIH/UT8656, AFPA00000000.1), *E. spinifera* (CBS 899.68, JYBY00000000.1), *R. mackenziei* (CBS 650.93, JYBU00000000.1), and *C. immunda* (CBS 834.96, JYBZ00000000.1) were assessed for the presence of genes required for this assimilation. The protein sequences of these strains clustered to 16,822 groups. No genes of the styrene degradation pathway were detected, but 16 clusters contained protein sequences required in the toluene pathway (Oelschlägel et al., 2018; Qu et al., 2019). *Exophiala spinifera* and *E. dermatitidis* differed significantly in the number of CYP450 genes, which were 123–133 and 61–72, respectively (Figure 4). In *E. spinifera*, dioxygenase (P340), which catalyzes the opening of the benzene ring, was missing in most of the strains. Several strains of both species lack β-carboxy-muconolactone hydrolase which is involved in degradation of the product of dioxygenase activity. Some variation was noted in the number of genes between strains of the same species, particularly in *E. spinifera*.

### Virulence Genes

A total of 813 genes associated with virulence and 18 virulence domains were detected in *E. dermatitidis* and *E. spinifera* (Table 4). The highest number (*n* = 458) was found in *C. immunda*, CBS 834.96, the lowest (*n* = 267) in *E. dermatitidis*, CBS 578.76. The number of virulence genes in *E. spinifera* ranged between 389 and 432, and in *E. dermatitidis* between 267 and 303. Of the genes that are possibly linked with brain invasion (*n* = 192), OG0012246 was consistently detected in *E. dermatitidis* and *R. mackenziei*. Hypothetical proteins OG0012600 and OG0012602 were lacking in some *E. dermatitidis*. None of these proteins was present in *E. spinifera.* Thirty-four genes have been suggested to play a role in bone invasion (Supplementary Table S-1). Of these, 3 were detected in *E. dermatitidis* and *E. spinifera*, of which linker histone H1/H4 and proline racemase were present in both species. In *E. spinifera* strains, proline racemase was replaced by proline oxidase (McKenna et al., 2014).

**TABLE 4:** Virulence domains detected in strains of *Exophiala spinifera* and *E. dermatitidis* (CBS accession numbers).

IPR/PFAM accession	Gene	*E. spinifera*					*E. dermatitidis*					
		101539	116557	123469	126013	131564	120473	109144	115663	132754	132758	578.76
IPR013912	Adenylate cyclase associated (CAP) C terminal	1	1	1	1	1	0	0	0	1	1	1
IPR013992	Adenylate cyclase associated (CAP) N terminal	0	0	0	0	0	1	1	1	0	0	0
IPR008441	Capsular polysaccharide synthesis protein	0	0	0	0	0	1	1	1	1	1	1
PF01302	CAP-Gly domain	4	4	3	4	4	3	4	4	3	3	3
PF00188	Cysteine-rich secretory protein family (CRISP)	5	5	5	5	5	5	6	5	5	5	5
PF06058	Dcp1-like decapping family	1	1	1	1	1	1	1	1	1	1	1
IPR001698	F-Actin capping protein beta subunit	1	1	1	1	1	1	1	1	0	0	1
IPR018814	Family of unknown function (DUF5427)	1	1	1	1	1	1	1	1	0	0	1
PF00012	Hsp70 protein	12	11	12	11	11	10	10	10	10	10	10
PF03291	mRNA capping enzyme	3	3	3	3	3	3	3	3	3	3	3
IPR019416	Nuclear cap-binding protein subunit 3	1	1	1	1	1	1	1	1	1	1	1
PF01255	Putative undecaprenyl diphosphate synthase	2	2	1	2	3	1	1	1	1	1	1
IPR019012	RNA cap guanine-N2 methyltransferase	1	1	1	1	1	1	1	1	1	1	1
PF11969	Scavenger mRNA decapping enzyme C-term binding	0	0	0	1	0	2	2	2	0	0	2
PF05652	Scavenger mRNA decapping enzyme (D) N-terminal	1	1	1	0	1	0	0	0	1	1	0
PF12658	Telomere capping CST complex subunit	1	1	1	1	1	1	1	1	0	0	1
PF00240	Ubiquitin family	12	11	12	11	12	9	10	9	10	9	9
IPR018814	Maintenance of telomere capping protein 1	0	0	0	0	0	0	0	0	1	1	0

Gene families PFam and IPR in the literature have been associated with virulence; strains were found to differ rather considerably in the presence of these genes (Table 4). *CAP* Gly domain, cysteine-rich secretory protein family, Hsp70 protein, mRNA capping enzyme and ubiquitin family were consistently present with higher copy numbers (Choudhary and Schneiter, 2012; Chen et al., 2019). From the virulence gene prediction, the adenylate cyclase associated (*CAP*) Gly-domain was present in both species, while capsular polysaccharide synthesis protein and *CAP* N-terminal were present only in some of the *E. dermatitidis* strains. *CAP* C-terminal was consistently present in *E. spinifera* but lacking in three *E. dermatitidis* strains.

Two *E. dermatitidis* strains, CBS 578.76 and CBS 120473, were isolated from human brain. The species is known to be neurotropic upon dissemination (Sudhadham et al., 2008). The strains were found to be close to each other, clustering with strain CBS 132758 collected from a dishwasher (Figure 1). There were 18,283 unique SNPs present in the strains from brain, associated with 1,144 genes. To reveal possible differences between *E. dermatitidis* isolates from human brain and from the environment, the protein sequences of the five *E. dermatitidis* genomes were clustered; 9,151 clusters were obtained. Among these, 876 clusters contained protein sequences that were only found in the two brain isolates CBS 578.76 and CBS 120473. The proteins in CBS 578.76 and CBS 120473 shared two domains, i.e., Myb-like DNA-binding domain and SANT/Myb domain (OG0012246) (Supplementary Table S-2). The former protein has morphogenetic roles (Wieser and Adams, 1995), while the latter enhances development and pathogenicity (Kim et al., 2014). Myb-like DNA-binding domains of RNA polymerase III transcription factor IIIB (TFIIIB) have a function in the assembly of DNA complexes and recruitment of RNA polymerase to the promoter (Kassavetis et al., 1997).

**FIGURE 5:**
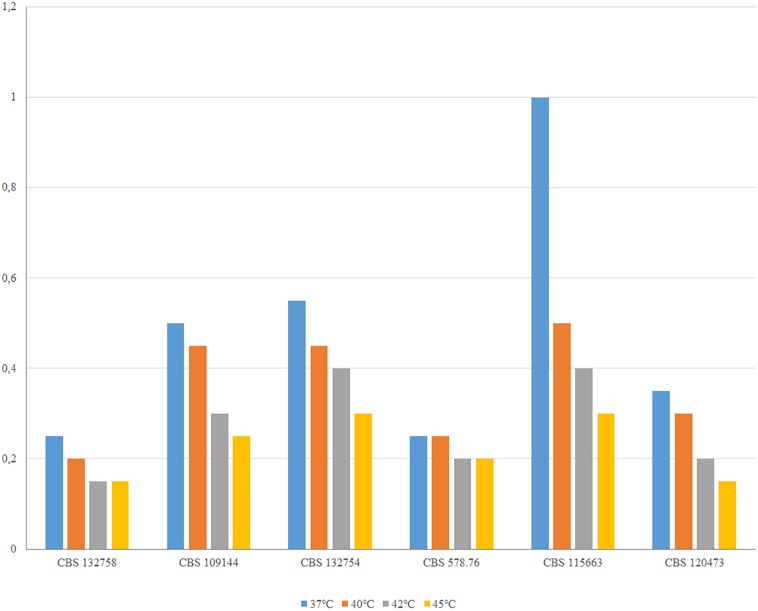
Thermotolerance of *E. phaeomuriformis* CBS 132758 and five *E. dermatitidis* strains measured after 2 weeks incubation on MEA medium.

*Exophiala phaeomuriformis* differs phenotypically from *E. dermatitidis* by its lower thermotolerance (de Hoog et al., 2019). This was confirmed in the analyzed set of strains: despite significant variation in growth velocity, *E. dermatitidis* strains all were able to grow at 45°C, while the maximum of *E. phaeomuriformis* was at 40°C (Figure 5).

## Discussion

From clinical data, i.e., the number of published cases and their severity (Sudhadham et al., 2008; de Hoog et al., 2019) it has been concluded that *E. dermatitidis* and *E. spinifera* are the most virulent species in the genus *Exophiala*. In contrast, *C. immunda* has never been observed in clinical settings, which may either be explained by low virulence or its rarity. Remarkably, this fungus was found to have the highest number (*n* = 458) of genes that have been associated in the literature with virulence (Fisher et al., 1986; Chamond et al., 2003; Xu et al., 2012; Isola et al., 2013; Gümral et al., 2014; Zhang et al., 2014; Gelli, 2018; Bernardes et al., 2019). *Exophiala dermatitidis* is a common environmental species occurring in the domesticated environment (Isola et al., 2013; Gümral et al., 2014) and thus has a higher chance to of infection; it has a significantly lower number (*n* = 267–403) of virulence-associated genes than all species compared, including the equally common environmental species *E. oligosperma* (*n* = 424).

All virulence genes in this study can be summarized into the following four major classifications, the capsular polysaccharide synthesis, the *CAP* associated proteins (including decapping enzyme and capping protein), the Hsp70 protein, and ubiquitin family. Notably, the capsular polysaccharide synthesis protein and *CAP* N-terminal is present in *E. dermatitidis* strains only. Both species share the same cysteine-rich secretory proteins (CRISP), which can induce an increase of leukocytes *in vivo*, stimulating the production of cytokines and eicosanoids (Xu et al., 2012). Expression of a human *CAP* superfamily member, the cysteine-rich secretory protein 2 (CRISP2), rescues the phenotype of yeast mutants lacking *Pry* function and purified CRISP2 binds cholesterol *in vitro*, indicating that lipid binding is a conserved function of the *CAP* superfamily proteins playing an important role in lipid and sterol metabolism. These genes are also significant in *Exophiala*.

*Exophiala dermatitidis* and *E. spinifera* are the only chaetothyrialean black yeasts that possess capsule-like EPS around juvenile cells, while these reportedly are absent from sibling species *E. phaeomuriformis* and *E. oligosperma*, respectively. These genes are known in *Cryptococcus*, where the microtubule-associated *CAP*-glycine protein (*Cgp1*) governs growth, differentiation, and virulence of *Cryptococcus neoformans* (Gelli, 2018). Phenotypically, differences are known in the shape of black yeast capsules, which are regular in *E. spinifera* and irregular EPS in *E. dermatitidis* (Song et al., 2017). In addition, *E. spinifera* loses capsule formation at 37°C, while most *E. dermatitidis* strains maintain this capacity (Song et al., 2017). Different from *Cryptococcus, Exophiala* species may lose this purported virulence when it is needed at elevated temperature during host invasion.

One of the predicted genes found in both *E. dermatitidis* and *E. spinifera*, Cysteine-rich secretory protein (*CRISP*), has an effect on potassium channels and inflammatory processes (Bernardes et al., 2019). Pathogen-Related Yeast (*PRY*) genes belong to a large *CAP* protein superfamily (i.e., *CRISP*, antigen 5, and pathogenesis related 1 proteins). The conserved *CAP* domain of *Pry1* is necessary and sufficient for lipid export and sterol binding (Choudhary and Schneiter, 2012).

There are two clusters belonging to the Myb-like DNA-binding domain and SANT/Myb domain only present in the neurotropic species *E. dermatitidis* and *R. mackenziei*, and absent from *E. spinifera* which was suggested to be somewhat osteotropic (Sudhadham et al., 2008). SANT/Myb-type genes are involved in conidiation of *Cochliobolus carbonum* (Zhang et al., 2014), while Myb-like DNA-binding protein that coordinates initiation of *Aspergillus nidulans* conidiophore development (Wieser and Adams, 1995). The virulence gene MYT3 is required for pathogenesis and sexual development in *Fusarium graminearum*. The Multiprotein Transcription Factor TFIIIB is linked to RNA polymerase III-transcribed genes indirectly through interaction with DNA-bound TFIIIC or directly through DNA recognition by the TATA-binding protein, in turn recruits RNA polymerase III to the promoter. It is a key transcription factor in *Saccharomyces cerevisiae* (Kassavetis et al., 1997).

The genes possibly associated to osteotropy were shared by *E. spinifera* and *E. dermatitidis*, suggesting that the different predilection of disseminated strains might rather be linked to absence of neurotropism in *E. spinifera* rather than presence of bone-related genes. Linker histone H1 is an essential component of chromatin structure, linking nucleosomes into higher order structures and is eventually replaced by H5. Histone proteins have central roles in both chromatin organization as structural units of the nucleosome and gene regulation. Proline racemase catalyzes the interconversion of L- and D-proline (Fisher et al., 1986; Chamond et al., 2003). Three strains deviated by having proline oxidase instead of racemase. The family also contains several proteins that remain hypothetical. FAD-linked oxidoreductase was detected in a clinical *E. spinifera* strain. This concerns a family of bacterial oxidoreductases with covalently linked FAD (McNeil and Fineran, 2013; Bogachev et al., 2018).

Hsp70 proteins is a class of molecular chaperones that are shared by both species. Studies in *Fusarium* showed that knockout of an ER lumenal Hsp70 homolog *FpLhs1* gene reduced growth, conidiation, and pathogenicity. *FpLhs1* is likely to act on the development and virulence by regulating protein secretion (Chen et al., 2019). The ubiquitin family is also essential in both species. The ubiquitin-proteasome system plays an essential role in the regulation of intercellular protein degradation, and the biosynthetic gene cluster for himeic acid A has been proven to be a ubiquitin-activating enzyme (E1) inhibitor in *Aspergillus japonicus* (Hashimoto et al., 2018).

Black yeasts in general display remarkably diverse lifestyles, with a predilection for extreme and toxic environments such as those rich in aromatic compounds or heavy metals, or with high temperatures, increased salinity, and scarcity of nutrients (Paolo et al., 2006; Prenafeta-Boldu et al., 2006; Zhao et al., 2010). Moreno et al. (2018) noted that chaetothyrialean black fungi are exceptionally rich in cytochrome P450 genes enhancing toxin management. No genes involved in the styrene pathway, but comparative analysis of *R. mackenziei* against the aromatic hydrocarbon-degrading fungus *C. immunda* (Moreno et al., 2018) revealed the presence of orthologs that resemble the published fungal toluene degradation pathway (Figure 4) via protocatechuate (Parales et al., 2008; Blasi et al., 2017). Toluene was proven to be initially oxidized to benzyl alcohol by a membrane-bound CYP in toluene-growing cells of the closely related black fungus *Cladophialophora saturnica* CBS 114326 (previously confused with *Cladosporium sphaerospermum*) (Luykx et al., 2003). *Exophiala spinifera* has twice as many CYP as *E. dermatitidis*, a species having the smallest genomes of all members of the family Herpotrichiellaceae sequenced thus far (Teixeira et al., 2017). On the other hand, in most *E. spinifera* strains, dioxygenases needed for the opening of benzene rings via protocatechuate are missing, while they are consistently present in *E. dermatitidis*. Notably, *p*-hydroxybenzoate hydroxylase is present in both species, while it was absent from the toluene-degrading black fungus *C. immunda* (Blasi et al., 2017). It may be surmised that in *E. spinifera* the CYP P450 genes have other functions than toluene degradation. Nascimento et al. (2017) found rich populations of *E. spinifera* in degrading coconut shells rich in esters and hydrocarbons, while *E. dermatitidis* is unambiguously associated with monoaromatic pollutants, alkanes and creosotes (Isola et al., 2013; Gümral et al., 2014). Although this ability may enhance neurotropism in *E. dermatitidis*, the human brain is unlikely as a natural habitat for the species, which should be considered as an opportunist rather than a pathogen, as is exemplified by a recently described hydrocarbon-degrading in the Antarctic (Zhang et al., 2019). Other indications were found in the adverse response of *E. spinifera* to elevated temperature, and variability of essential cytochromes. A possible additional gene promoting neurotropism is OG0012246 which was consistently detected in *E. dermatitidis* and *R. mackenziei* but was absent from *E. spinifera*.

## Data Availability Statement

The datasets generated for this study can be found in the GenBank Accessions:: JAAAJH000000000, JAAAJG000000000, JAAAJF000000000, JAAAJE000000000, JAAAJD000000000, JAAABF000000000, JAAABG000000000, JAAABH000000000, JYBY00000000, JYCA01000000, JAAAJI000000000, WXYG00000000, JAAAJK000000000, JAAAJJ000000000, JAAAJM000000000, JAAAJL000000000, AFPA00000000.1, and JYBU00000000.1.

## Author Contributions

RL and GH designed the experiments and supervised the data analysis. YS performed the experiments and wrote the manuscript. NS, VW, and DV analyzed the data. LM and VV provided technical support. All authors discussed the results and commented on the manuscript.

## Conflict of Interest

The authors declare that the research was conducted in the absence of any commercial or financial relationships that could be construed as a potential conflict of interest.

## Footnotes

1 https://www.bioinformatics.babraham.ac.uk/projects/fastqc/

2 https://sourceforge.net/projects/bbmap/

3 https://broadinstitute.github.io/picard/

4 http://vcfannotator.sourceforge.net/

5 http://www.ebi.ac.uk/interpro/
